# FOSTERING CONSISTENT AND ENGAGING CARE FOR PEOPLE WITH DEMENTIA IN LONG-TERM CARE

**DOI:** 10.1093/geroni/igad104.1068

**Published:** 2023-12-21

**Authors:** Jennifer Morgan, Candace Kemp, Andrea Hill, Alexis Bender

**Affiliations:** Georgia State University, Atlanta, Georgia, United States; Georgia State University, Atlanta, Georgia, United States; Georgia State University, Atlanta, Georgia, United States; Emory University, Atlanta, Georgia, United States

## Abstract

Maintaining a stable and qualified direct care workforce in long-term care organizations has become increasingly difficult as shortages intensify, turnover increases, and the use of agency staff remains at high levels. Efforts to improve this situation need to include high quality training that is incorporated into onboarding, ongoing continuing education, and infused into the culture of the organization. Using data from our ongoing NIA-funded study, “Meaningful Engagement and Quality of Life among Assisted Living Residents with Dementia,” we describe the competencies used by direct care workers (DCWs) with exceptional capacity, or ability and resources to engage people with dementia, and the factors that promote or limit the development and use of these competencies. Focal residents with dementia (N=66) were followed at eight diverse assisted living communities for one year and 53 DCW were interviewed across three waves of data collection. Interview transcripts and fieldnotes from more than 2000 hours of observation were analyzed using a grounded theory approach. Organized by four engagement approaches: knowing the person, connecting with and meeting people where they are, being in the moment, and viewing all encounters as opportunities, we identified competencies and the multiple intersecting factors which impact meaningful engagement and competency development including community staffing, consistent assignment, scheduling, DCW training, DCW tenure, and community resources. Implications for practice, which emphasize the elaboration of specific competencies, have the potential to inform the development of DCW training, the promotion of supportive work environments, and social policy that support person-centered dementia care.

